# Past Traumatic Life Events, Postpartum PTSD, and the Role of Labor Support

**DOI:** 10.3390/ijerph20116048

**Published:** 2023-06-04

**Authors:** Anna Suarez, Vera Yakupova

**Affiliations:** Department of Psychology, Lomonosov Moscow State University, Moscow 119991, Russia; vera.a.romanova@gmail.com

**Keywords:** postpartum PTSD, past trauma, child abuse, sexual assault, physical assault, perinatal loss, birth support

## Abstract

The aim of this study was to investigate the association of postpartum post-traumatic stress disorder (PP-PTSD) symptoms and subjective rates of traumatic birth experience with past traumatic life events (physical and sexual assault, child abuse, perinatal loss, previous traumatic birth experience, and the cumulative traumatic experience). A sample of Russian women (*n* = 2579) who gave birth within the previous 12 months, filled in a web-based survey, where they reported demographic and obstetric characteristics and past traumatic experiences, evaluated their birth experience (0 = not traumatic, 10 = extremely traumatic), and completed the City Birth Trauma Scale (CBiTS). We found that PP-PTSD symptoms were higher among women who previously experienced physical (F = 22.02, *p* < 0.001) and sexual (F = 15.98, *p* < 0.001) assault and child abuse (F = 69.25, *p* < 0.001), with only associations with child abuse (F = 21.14, *p* < 0.001) remaining significant for subjective rates of traumatic birth experience. Perinatal loss and previous traumatic birth showed moderate but inconsistent effects. Support during labor did not have a buffering effect for participants with past traumatic experiences but showed a universally protective effect against PP-PTSD. Trauma-informed practices and allowing women to have a supportive birth team of choice during childbirth are promising avenues to minimize the incidence of PP-PTSD and improve the childbirth experience for all women.

## 1. Introduction

While childbirth can be a deeply meaningful and transformative experience for women, it is also associated with a significant risk of trauma. The prevalence of traumatic childbirth experiences varies widely, with studies showing that anywhere from 3% to 45.5% of women may perceive their childbirth as traumatic [1,2,3]. Furthermore, research suggests that 3.1% to 43% of women may go on to develop postpartum post-traumatic stress disorder (PP-PTSD) [4,5,6]. It is characterized by re-experiencing a traumatic event through nightmares, flashbacks, and intrusive memories; avoiding stimuli associated with the traumatic event; hyperarousal; negative cognitions and mood; and significant impairment and distress [7]. These symptoms significantly reduce women’s quality of life and can last for months following childbirth or become chronic [8,9]. Qualitative studies showed that some women had been suffering from this disorder for as long as 18 years after childbirth [10]. PP-PTSD may have further devastating ripple effects on women’s emotional, physical, and social wellbeing [11] and compromise breastfeeding [12,13,14] and mother–infant bonding [11,15,16]. Moreover, both in case studies and qualitative research, women reported the adverse impact of PP-PTSD on relationships with their partners, including blame for the events during childbirth, disagreements, and sexual dysfunction [11,17], the latter often caused by an intense fear of getting pregnant again. 

Thus, addressing the risk factors for PP-PTSD for timely and accurate support and prevention is vital. Some of the strongest predictors of PP-PTSD and traumatic birth experience relate to the intrapartum risk factors, such as obstetric emergencies and infant complications [18], medical interventions [18,19,20], preterm birth [21], instrumental birth with forceps or a vacuum [4,18], emergency cesarean birth [4,20,22], obstetric violence [20,23,24,25], and insufficient support from caregivers and partner during labor and delivery [18,19,22,24,26]. Furthermore, the postpartum period presents additional risk factors such as maternal and neonatal complications [18,25], hospital readmission [23], postpartum depression [22], and women’s dissatisfaction with social support [27]. 

Sadly, these groups of factors usually occur unexpectedly, which limits the opportunities for prevention. However, there is an increasing number of studies indicating that there are pre-existing risk factors for developing PP-PTSD that could be addressed during pregnancy or even before conception. They include a history of psychiatric disorders [18,22,28], depression and poor physical health during pregnancy [22], and previous traumatic experiences [18,19,22]. 

Indeed, PP-PTSD can be conceptualized both as the first-time onset related solely to childbirth in the absence of prior PTSD symptoms or any predisposing factors and as a pre-existing PTSD after previous traumatic events that are reactivated by childbirth [4,5]. The study by Oliveira et al. [29] found that among women who developed PP-PTSD, 30.2% reported experiencing sexual abuse during their childhood. Additionally, 92.5% of these women reported experiencing psychological abuse, and 45.0% reported experiencing physical abuse from their partners during pregnancy. Seng et al. [30] further found that pregnant women with PTSD were five times more likely to have a history of completed childhood rape than those without PTSD. Similarly, multiple studies indicate that women with a history of sexual assault or childhood sexual abuse are more likely to develop PP-PTSD [5,18,31,32]. As the WHO estimates that globally 1 in 5 women were sexually abused at the age of 0–17 years [33] and 1 in 3 had experienced intimate partner violence or non-partner sexual violence in their lifetime [34], the scope of the potential risk of developing PP-PTSD is massive. 

Furthermore, it is estimated that up to 25% of pregnancies end with a perinatal loss, and perinatal grief is an important predictor of PTSD symptoms [35,36]. In a study among 97 Israeli women, the authors observed that up to 33% of women experienced symptoms of PTSD [37], while in a prospective study from the Netherlands, the prevalence of PTSD was 25% [38]. However, the latest meta-analysis did not confirm the association between perinatal loss and post-traumatic stress outcomes [39]. Nevertheless, as 50–80% of women who experience perinatal loss conceive again [35], pregnancy after perinatal loss is a common event, and past experience of PTSD symptoms may affect mental health during the subsequent pregnancy and childbirth. Indeed, a Spanish study by Ordóñez et al. [40] found that among 115 women who had experienced a previous perinatal loss, there was a higher risk of developing PTSD symptoms during the subsequent pregnancies, which may have acted as a trigger, particularly following multiple gestational losses. A scoping review also showed that women who were having children after the perinatal loss experience were particularly vulnerable to developing PTSD [41]. Fewer studies explored the prevalence of PP-PTSD among women who reported their previous birth experience as traumatic. However, the limited review articles indicate that, in fact, women with previous birth trauma are at higher risk of developing PP-PTSD [5,18].

A prospective cohort study of 933 women from Australia showed that participants who developed PTSD after childbirth and those who did not differ in fourteen characteristics, of which seven were related to past traumatic events in their lives [1]. Thus, the authors concluded that the most significant predictive factor for developing PP-PTSD was a previous traumatic experience. Therefore, it is essential to explore the factors which make women with past traumatic experiences more vulnerable to the risk of developing PP-PTSD and what practices could moderate these effects.

Support during labor has been consistently associated with more positive birth experiences [42,43] and a lower risk of PP-PTSD [44]. According to a Greek study, women with insufficient perinatal support from their partners were more likely to suffer from PTSD or PTSD symptoms after a cesarean birth than women who received adequate support from their spouses [45]. Furthermore, Handelzalts et al. [46] found that participants who were supported by their partners and a second companion had fewer PP-PTSD symptoms than women who were accompanied only by their partners. Accordingly, several Russian studies showed that although there were no significant direct associations between support during labor and PP-PTSD, support from a partner, doula, or private midwife during childbirth was associated with lower rates of obstetric violence, fewer medical interventions, and higher birth satisfaction, which may indirectly mitigate the risk of PP-PTSD [20,24,47].

While the prevalence of clinically significant PP-PTSD is estimated at 15% in Russian women [20], to our knowledge, no previous studies focused on the past traumatic event as a risk factor for PP-PTSD in the Russian population. At the same time, WHO reported that in a sample of 1580 young students, adverse childhood experiences (ACE) were highly prevalent, with 84.6% of participants having disclosed at least one ACE [48]. Furthermore, according to an independent survey of 17,000 people, where 99% of respondents were women, 40% of participants experienced some form of sexual abuse [49]. The Russian non-governmental organization “Light in Hands” also reports approximately 16 neonatal deaths, 32 stillbirths, and 320 losses due to miscarriage and termination of pregnancy for medical reasons daily in Russia [50]. Thus, the prevalence of women with past traumatic life events is high in Russia; however, the effects of these events on the risk of developing PP-PTSD remain unknown.

Therefore, the main objective of this study was to investigate the association of PP-PTSD symptoms and subjective rates of traumatic birth experience with past traumatic life events, namely physical and sexual assault, child abuse, perinatal loss, and previous traumatic birth experience, as well as the cumulative traumatic experience in a Russian sample. Furthermore, we explored the association between PP-PTSD symptoms and subjective rates of traumatic birth experience and support during labor. Finally, the aim of this study was to examine whether support during labor buffered the effects of past traumatic life events so that it had a stronger protective effect among women with previous traumatic experiences in comparison to those without such experience. We expect to see that women who experienced any type of traumatic life events in the past have both higher symptoms of PP-PTSD and rate their childbirth experience as more traumatic. We further hypothesize that having a support person present will be associated with both lower symptoms of PP-PTSD and subjective rates of the traumatic birth experience. Support will also moderate the association between past traumatic experience and PP-PTSD so that there will be a significantly stronger effect of support for trauma survivors than for women without past traumas. 

## 2. Materials and Methods

### 2.1. Participants

Data for this cross-sectional study were collected from May to September 2022. Women received invitations to participate in the web-based survey via social media (relevant Instagram, Facebook, and VK communities and perinatal health professional pages), antenatal classes, and classes for new parents as well as from the doctors and midwives in maternity hospitals and healthcare clinics. In total, 2954 women responded to the questions, and 2579 fulfilled the inclusion criteria (they were 18 years old or above, gave birth within the previous 12 months to live-born children, could read and type in Russian, and childbirth took place in Russia).

### 2.2. Ethical Considerations

This present study was approved by the Ethical Committee of the Russian Psychological Society at Lomonosov Moscow State University (No: 345/2019). All participants signed the informed consent forms using the online tool Testograph prior to filling in the survey. The study design and procedures are compliant with the Declaration of Helsinki.

### 2.3. Measures

#### 2.3.1. Demographic and Obstetric Variables 

The survey included questions about the participant’s age at the time of childbirth, highest achieved level of education (primary/secondary/tertiary), marital status (married/in relationship/single), and socioeconomic status (SES) in comparison to others in the current region of residence (low/middle/high).

Respondents also answered questions regarding pregnancy and childbirth, such as parity, gestational age at childbirth, time since the childbirth, and mode of birth (vaginal/assisted vaginal/emergency cesarean/planned cesarean). Furthermore, women indicated whether they had a support person present at their childbirth and, if yes, who accompanied them (no support/partner/doula or private midwife/partner and doula or private midwife).

#### 2.3.2. Birth-Related Trauma and Past Traumatic Experience

Women were further asked to rate how traumatic they found their latest childbirth experience overall, on a scale from 0 (‘Not at all traumatic’) to 10 (‘Extremely traumatic’).

Next, there were questions asking to indicate whether the participants had ever experienced pregnancy loss, such as miscarriage or stillbirth, and, in case that they had given birth before, whether they considered their previous childbirth traumatic. 

Furthermore, women were presented with a list of other potentially very stressful and traumatic events (Serious, life-threatening illness/Physical assault/Sexual assault/Military combat or lived in a war zone/Child abuse/Accident/Natural disaster/Other trauma). They were asked to tick the boxes next to the events they had experienced or witnessed at some point in their lives. For the purposes of this study, we focused on the traumatic experiences that were previously linked to perinatal mood and anxiety disorders, namely physical assault, sexual assault, and child abuse, as well as the cumulative traumatic experience that was calculated as the sum of traumatic events, potentially ranging between 0 = no trauma in the past and 8 = exposure to all types of traumatic events. 

#### 2.3.3. Postpartum Depression (PPD) 

The Russian version (Cronbach’s α = 0.87) [51] of the Edinburgh postnatal depression scale (EPDS) [52] was used to estimate the symptoms of PPD. It is a 10-item questionnaire scale rated on a 4-point Likert scale, ranging from 0 to 3, which indicates how the mother has felt during the previous week. A cut-off score of 10 was used to identify women at risk of developing clinically significant symptoms of PPD [52]. 

#### 2.3.4. Postpartum PTSD (PP-PTSD) 

We used the City Birth Trauma Scale (CBiTS) [53] to assess PP-PTSD symptoms according to the Diagnostic and Statistical Manual-version 5 (DSM-5) [7]. 

It is a self-report 31-item questionnaire, with 29 questions mapping onto DSM-5 diagnostic criteria and 2 questions relating to DSM-IV criteria [53]. Scores are calculated by the addition of results of questions 3–22; each question is scored on a Likert-type scale ranging from 0 (‘not at all’) to 3 (‘5 or more times’), with a total score range of 0–60. Higher scores represent greater levels of clinical symptomatology. This tool covers four clusters of symptoms according to DSM-5: ‘re-experiencing’ symptoms (5 questions), ‘avoidance’ symptoms (2 questions), ‘negative mood and cognitions’ symptoms (7 questions), and ‘hyperarousal’ symptoms (6 questions). In addition to these, there are two questions relating to the impact of the symptoms, dissociative symptoms such as feeling detached from reality, and onset (before childbirth/in the first 6 months following birth/later than 6 months after giving birth) and duration (less than 1 month, 1–3 months, more than 3 months) of symptoms. As deficits need to be present in all domains to warrant clinically relevant symptoms of PTSD, women can score very highly but not reach the threshold for diagnosis. Overall, the CBiTS shows high reliability and good psychometric properties, with high internal consistency (Cronbach’s α = 0.92) in the original study [53] and validation in multiple international studies [54,55,56], including the Russian version (Cronbach’s α = 0.90) [20].

#### 2.3.5. Covariates

All models were adjusted for the following maternal and birth-related characteristics previously associated with PPD and PP-PTSD as covariates: maternal age at the time of childbirth, level of education, marital status, socioeconomic status (SES), and previously diagnosed mental disorders as well as gestational age at birth, parity, time since the childbirth, and mode of birth [20,24,57].

#### 2.3.6. Statistical Analyses

Spearman’s correlation coefficient was used to estimate the relationship between PP-PTSD and PPD symptoms and the subjective rates of traumatic birth experiences.

Multiple linear regression analysis examined the association between PP-PTSD symptoms as well as the subjective rates of traumatic birth experience and cumulative past traumatic experiences.

We explored the association of the PP-PTSD symptoms and the subjective rates of traumatic birth experience with the categorical predictors (physical assault, sexual assault, child abuse, perinatal loss, previous traumatic birth experience, and support during labor) using generalized linear models.

Logistic regression analysis was performed to assess the association between the clinically significant PP-PTSD (according to DSM-5) and the cumulative past traumatic experiences and support during labor. 

Finally, we tested whether support during labor moderated the association between past trauma and PP-PTSD symptoms and subjective rates of traumatic birth experience. In order to conduct that, we first stratified the sample by the presence of traumatic events in the past (any trauma (y/n), physical assault (y/n), sexual assault (y/n), child abuse (y/n), perinatal loss (y/n), and previous birth experience (none/not traumatic/traumatic) and performed multiple linear regression adjusted for covariates with support during labor (y/n) variable as the predictor. Next, we performed the interaction analyses using multiple linear and logistic binary regression, where the models were adjusted for the covariates and past traumatic experience and support variables’ main effects and the interaction component of these two variables as the predictor in order to explore whether the discovered differences were statistically significant. 

The level of significance was set to α = 0.05. All analyses were performed using SPSS 27 software [58].

## 3. Results

The demographic, childbirth, and trauma-related characteristics of participants are presented in Table 1. It shows that most participants were highly educated (91.1%), married (91.7%), and had average income in comparison to other families from their region (66.2%). For most participants, it was their first child (63.1%), and they had a vaginal birth (73%) and had no support person present at birth (58%).

### 3.1. Prevalence of Postpartum Depressive and PTSD Symptoms and Past Traumatic Experience

Table 1 shows that among the participants, 37.5% had clinically significant depressive symptoms (EPDS scores > 10), and 20.5% of women fulfilled all the DSM-5 diagnostic criteria for PTSD (according to CBiTS scores). PPD and PP-PTSD symptoms were highly correlated in our sample (Pearson correlation = 0.66, *p* < 0.001). Both PPD (Pearson correlation = 0.34, *p* < 0.001) and PP-PTSD (Pearson correlation = 0.46, *p* < 0.001) also significantly correlated with the subjective rates of birth trauma, although to a lesser extent.

More than half of the participants (53.8%, *n* = 1388) indicated that they experienced or witnessed at least one traumatic event in the past. Furthermore, 23.1% of women experienced perinatal loss, and 20% said they had a traumatic childbirth experience previously. 

### 3.2. Postpartum PTSD and Past Traumatic Experiences

**Figure 1:**
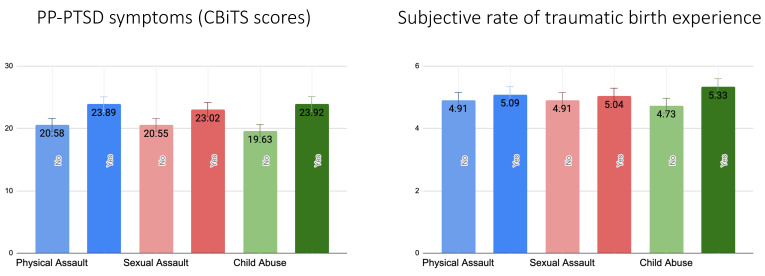
A bar chart reflecting mean values with standard error bars of PP-PTSD measured as continuous CBiTS scores and subjective rates of traumatic birth experience in relation to past traumatic experience (physical assault, sexual assault, child abuse). All values are adjusted for maternal age at the time of childbirth, level of education, family status, SES, history of mental disorders, gestational age at birth, time since childbirth, and mode of birth.

Figure 1 shows that after adjustment for covariates symptoms of PP-PTSD were significantly higher for those who experienced physical assault (F = 22.02, *p* < 0.001), sexual assault (F = 15.98, *p* < 0.001), and child abuse (F = 69.25, *p* < 0.001). However, in relation to subjective rates of traumatic birth experiences, the association remained significant only for those who reported child abuse (F = 21.14, *p* < 0.001) in the past but not physical (F = 1.12, *p* = 0.29) or sexual assault (F = 0.78, *p* = 0.38). 

Furthermore, there was a significantly higher risk of having PP-PTSD symptoms compatible with DSM-5 PTSD diagnosis if the participants had previously experienced child abuse (OR = 1.60, 95% CI 1.24; 2.05, *p* < 0.001) but not physical (OR = 1.11, 95% CI 0.78; 1.57, *p* = 0.56) or sexual assault (OR = 1.28, 95% CI 0.94; 1.74, *p* = 0.11).

**Figure 2:**
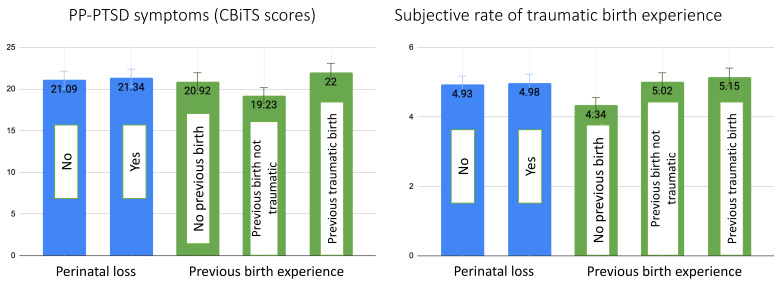
A bar chart reflecting mean values with standard error bars of PP-PTSD measured as continuous CBiTS scores and subjective rates of traumatic birth experience in relation to past traumatic experience perinatal loss and previous traumatic birth experience. All values are adjusted for maternal age at the time of childbirth, level of education, family status, SES, history of mental disorders, gestational age at birth, time since childbirth, and mode of birth.

Figure 2 shows that there were no significant associations between the participants who experienced perinatal loss and those who did not either in terms of PP-PTSD symptoms (F = 0.33, *p* = 0.57) or subjective rates of traumatic birth experience (F = 0.18, *p* = 0.67). Similarly, we did not find statistically significant associations between previous traumatic birth experiences and the subjective rates of current traumatic birth experiences (F = 2.30, *p* = 0.10) (Figure 2). Contrarily, PP-PTSD symptoms were lowest for those who already gave birth previously, and that experience was not traumatic; they were, on average, more than 1.5 points higher for those who gave birth for the first time in this present study, and, finally, the highest scores were among the participants whose previous birth experience was also traumatic (F = 10.04, *p* < 0.001) (Figure 2). 

However, the risk of having a clinical PP-PTSD diagnosis was significantly higher both in cases where the women had a perinatal loss (OR = 1.29, 95% CI 1.02; 1.64, *p* = 0.032) or previous traumatic birth (OR = 1.49, 95% CI 1.05; 2.11, *p* = 0.025) experience.

Table 2 shows that after adjustment for covariates, the cumulative traumatic experiences were significantly associated with both PP-PTSD symptoms and subjective rates of the traumatic birth experience. Furthermore, these participants had an over 25% higher risk of presenting symptoms compatible with PP-PTSD diagnosis with each additional traumatic event they experienced in the past. 

### 3.3. Postpartum PTSD and Support during Labour 

Table 2 demonstrates that women who gave birth in the presence of a support person scored lower on both the PP-PTSD scale and the subjective scale of traumatic birth experience as well as had a significantly lower risk of having a clinical PP-PTSD diagnosis (Table 2). 

**Figure 3:**
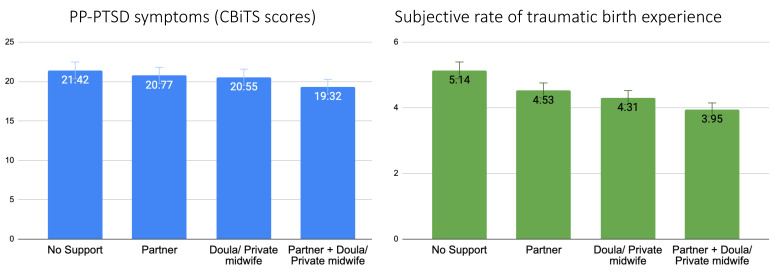
A bar chart reflecting mean values with standard error bars of PP-PTSD measured as continuous CBiTS scores and subjective rates of traumatic birth experience in relation to the mode of support the participants had during labor. All values are adjusted for maternal age at the time of childbirth, level of education, family status, SES, history of mental disorders, gestational age at birth, time since childbirth, and mode of birth.

After adjustment for covariates, the highest PP-PTSD scores were among the participants who gave birth without support, slightly lower and almost equal scores were among women who gave birth in the presence of a partner or a doula/private midwife, and the lowest scores were among those who gave birth in the presence of both a partner and a doula/private midwife (F = 3.09, *p* = 0.026) (Figure 3). Figure 3 further shows that women who subjectively rated their childbirth experience as most traumatic had no support person present during labor, whereas women who rated their childbirth experience as least traumatic were supported by both their partners and a hired professional (doula or private midwife) (F = 22.68, *p* < 0.001).

### 3.4. Moderation Analysis 

To estimate whether support during labor had a stronger effect among those who had traumatic experiences in the past in relation to PP-PTSD and subjectively rated traumatic birth experiences, we performed multiple regression analyses in the subsamples stratified by the presence of trauma in the past. Table S1 shows that the effects of the presence of a support person during labor on the PP-PTSD symptoms were more significant among women who reported at least one traumatic event in the past, who experienced perinatal loss, or previous traumatic childbirth. However, those associations were attenuated or more significant among those with no past trauma in relation to physical or sexual assault and child abuse (Table S1). Furthermore, the interaction analyses revealed that these differences between the subsamples were not statistically significant (*p*-values for all >0.066, data not shown). 

In the analysis with subjective rates of traumatic birth experience as the outcome, we found that support during labor was statistically significant in the subsamples with and without past trauma (Table S1), and the interaction analyses showed no statistical significance (*p*-values for all >0.50, data not shown). 

## 4. Discussion

To our knowledge, this present work is the first to explore the association between traumatic birth experience and past traumatic life events that include physical assault, sexual assault, child abuse, perinatal loss, previous traumatic birth experience, and cumulative trauma in one study. Furthermore, we focused on the role of support during labor and its types, its association with PP-PTSD, and the potential buffering role for the participants with past traumatic experiences. We found that PP-PTSD symptoms were significantly higher among women with a history of physical and sexual assault, child abuse, and past traumatic birth experience, but not perinatal loss. Subjective rates of traumatic birth experience were significantly higher only among women with child abuse experience. In accordance with our hypotheses, we showed that support during labor was associated with lower PP-PTSD symptoms as well as subjective rates of traumatic birth experiences. However, contrary to our expectations, there was no buffering effect of the presence of a support person for women with past traumatic experiences.

While PP-PTSD symptoms correlated significantly with subjective rates of traumatic birth experience in our study, this association was weaker than with PPD symptoms. It is in line with previous reports of the high comorbidity of PPD and PP-PTSD in Russia [20] and globally, with some studies reporting that 90% of women with PP-PTSD also experience higher symptoms of PPD [59]. It may indicate that women with PP-PTSD can be identified and treated more frequently by clinicians due to the more common monitoring of PPD and the shared symptomatology of the two disorders, whereas women who report their childbirth experience as traumatic without presenting symptoms of either PP-PTSD or PPD, may not receive necessary support despite their real suffering. At the same time, it corroborates a previous report regarding longitudinal trajectories of PP-PTSD, which suggests that traumatic birth does not necessarily result in PTSD, and if it does develop, a substantial proportion of women show resilience and can recover within the next 3–12 months [60]. As we collected the data from women who gave birth within the past 12 months, some of the participants who rated the birth experience as traumatic but did not have elevated symptoms of PP-PTSD might have already recovered by the time of the participation. Thus, further longitudinal studies with multiple data collection points across the first year after childbirth are warranted to evaluate the trajectories of PP-PTSD, PPD, subjective rates of traumatic birth experiences, and their covariance.

We further found that PP-PTSD symptoms were higher among women who experienced physical and sexual assault in the past, while there were no such associations with subjective rates of traumatic birth experiences. These results contribute to the line of studies that conceptualize PP-PTSD as pre-existing PTSD, which is reactivated by childbirth experience [4,5], whereas subjective perceptions of birth as traumatic may be related to the events of labor and birth. On the other hand, both PP-PTSD symptoms and subjective rates of traumatic birth experience were higher among participants with a history of childhood abuse. Multiple reviews and meta-analyses have shown that, indeed, childhood adversity is one of the most significant risk factors for developing PP-PTSD [5,19]. Neurobiological research suggests that childhood abuse is strongly associated with dysregulation of the hypothalamic–pituitary–adrenal (HPA) axis, which has long-term effects on stress response [61]. Moreover, studies have identified multiple changes in the brain structures in individuals with a history of childhood maltreatment [62,63]. Thus, prolonged maternal childhood abuse and neuroendocrine abnormalities associated with it can contribute to birth complications such as preterm delivery [64] and low birth weight [65], which, in turn, present an additional risk for PP-PTSD [21]. At the same time, symptoms of depression and post-traumatic stress may impede a sense of self-efficacy in pregnant women with a history of early life trauma and abuse and impair the process of communicating their choices to obstetricians. As a result, these women are subjected to more invasive obstetric exams and interventions than is typically recommended [66], which means that, currently, women who are most prone to experience acute distress during obstetric procedures may also be least likely to express their distress to healthcare providers. Therefore, new protocols among healthcare professionals should be implemented so that women with a history of childhood abuse can be identified and receive additional support already during pregnancy, as they are at particularly elevated risk for traumatic birth experiences, even in the absence of a PTSD diagnosis. 

We also show that past traumatic events act cumulatively such that the more traumas women reported, the higher were both PP-PTSD symptoms and subjective rates of traumatic birth experiences. Similarly, exposure to more traumatic events was associated with a higher risk of developing PTSD following childbirth in French [67], Canadian [68], and US studies [69]. Investigation of potential biological mechanisms of the effects of cumulative trauma exposure in community samples of pregnant women in Greece, Spain, and Perú revealed that trauma load was negatively associated with hair cortisol concentrations and positively associated with symptoms of depression and anxiety, supporting previous observations that trauma exposure exerts long-lasting effects on the body’s stress response system [70].

Interestingly, we found no significant associations in both PP-PTSD symptoms and subjective rates of traumatic birth experience with perinatal loss experience. However, women were at 1.29 times higher risk of having PP-PTSD symptoms compatible with DSM-5 PTSD diagnosis if they experienced perinatal loss in the past. These results contribute to the contradictory evidence of the significance of perinatal loss on maternal mental health in subsequent pregnancies and the postpartum period [39,40]. Perinatal loss may be experienced very differently, ranging from being an unfortunate medical event to the feeling of deep loss and grief. Nevertheless, a recent scoping review found that up to 60% of parents met the criteria for PTSD immediately after perinatal loss, with many of them experiencing persistent symptoms of PTSD for years following their loss experience [41]. Similar to our study, Turton and colleagues [71] showed that stillbirth was associated with a lifetime PTSD prevalence of 29% and 20% prevalence in subsequent pregnancies. Thus, the mixed findings might indicate that perinatal loss is a complex event with high variability in individual and cultural responses to it. However, if it is followed by PTSD immediately after the loss experience, it presents a significant risk factor during the subsequent pregnancy and postpartum.

Our work is among the few studies that address the consequences of previous traumatic birth experiences for subsequent childbirth and postpartum mental health. We found that it was significantly associated with PP-PTSD but not the subjective rates of traumatic birth experience. Intriguingly, symptoms of post-traumatic stress following childbirth were almost as high in first-time mothers as in those whose previous birth was traumatic. It might indicate that it is not the previous traumatic birth that elevates the risk for a repeated traumatic birth, but, rather, a positive childbirth experience is a protective factor against PP-PTSD after following births. Indeed, higher birth satisfaction has been associated with lower rates of PP-PTSD in Turkey [72], Croatia [73], and England [74]. Susan Ayers has emphasized that while the healthcare and research culture has been largely focused on the investigation of risks and their avoidance, it is important to shift the perspective to the exploration of protective factors that may play open avenues for preventive measures [75]. Nevertheless, midwives and other healthcare professionals should be aware of the potential risks of previous traumatic birth experiences for postpartum mental health in subsequent births and encourage women to grieve their prior traumatic births to help remove the burden of their invisible pain, as subsequent childbirth has the potential to either heal or retraumatize women [76].

We also show that support during labor has been a strong protective factor in our study: women who had at least one support person present at childbirth had a significantly lower risk of PP-PTSD, lower scores of PP-PTSD symptoms, and lower rates of subjective traumatic birth experiences. There are only two previous studies of PP-PTSD and support during labor in Russia, where, contrary to our current findings, there was no direct association between the presence of at least one birth companion and PP-PTSD [20,24]. However, it was associated with lower rates of cesarean births, obstetric violence, and medical interventions, which are all significant risk factors for developing PP-PTSD. It is important to note that in the previous studies, the sample sizes were substantially smaller than in this present study, and the participants mainly came from the big Russian cities with a population of over 1 million citizens, while here, they come from a variety of locations in Russia. Thus, this present work might reflect a more precise picture of the importance of support during labor and its direct protective effects against developing PP-PTSD. Moreover, these results contribute to a substantial body of evidence showing that continuous non-medical support during labor is associated with overall better childbirth outcomes, such as lower rates of cesarean births and negative childbirth experience reports [77] and lower rates of traumatic birth experiences, indicated both by systematic reviews [27,67] and prospective studies [78]. 

Furthermore, we show that both PP-PTSD symptoms and subjective rates of traumatic birth experience were lowest among women who had both their partner and a doula or private midwife present at birth, which is supported by a previous study where authors show similar results as our findings, with higher PP-PTSD symptoms among women with a single companion compared to those with two or more companions [46]. Thus, allowing more than one person to accompany women during childbirth may be a simple and cost-effective approach for providing support in all birth settings and minimizing the incidence of PP-PTSD.

Finally, we hypothesized that support during labor had a stronger protective effect among women with previous traumatic experiences in comparison to those without such experience. Contrary to the study from the UK, where support during childbirth was buffering against traumatic birth events, with the strongest effects among women with previous histories of trauma or abuse [78], we did not see such effects among women with any type of past trauma. Instead, we see its universal importance for all women, particularly in relation to the subjective perception of the birth experience. Importantly, in the study of Ford and Ayers [78], it was midwives’ and healthcare professionals’ support that demonstrated the buffering effect against PP-PTSD, while in our study, we focused on the non-medical continuous support by a partner and/or a paid female professional, i.e., a doula or a private midwife. Midwives and clinicians are the ones who have the responsibility, right, and opportunity to perform medical procedures; therefore, their support during labor directly affects the childbirth experience. Furthermore, women are particularly vulnerable during labor, highly dependent on the actions of midwives and other hospital staff members. Both physical and emotional conditions have been shown to be highly triggering for bringing back memories of abuse during labor [31,79]. Yet, depending on the birth culture in a particular country or even a particular hospital, it is not always possible to choose the medical team and, thus, rely on the trauma-informed training and support of the midwives. At the same time, it is becoming more popular and accessible to have a companion during labor, thus allowing women to choose persons(s) whom they trust and feel safe with. Sadly, although the WHO strongly recommends supporting women in having a chosen companion during labor [80], in many countries, including Russia, these recommendations are often dismissed. For example, despite the presence of a birth partner becoming a legal right since 2012 [81], small maternity care hospitals can still restrict their presence during labor due to the absence of individual wards. Moreover, an opportunity for continuous individual support by a doula or a privately hired midwife is possible only if the woman pays both for a doula/private midwife service and for the contract with the hospital to have this option included in her childbirth plan, with each maternity hospital reserving the right to decline such a possibility as it’s not guaranteed by the law [82]. Therefore, the global legalization of doula communities within the state healthcare system could provide the continuous support during labor recommended by WHO [83], which is a promising way to support all women’s perinatal mental health.

## 5. Strengths and Limitations

The strengths of our study include substantial sample size, the study design, use of validated questionnaires, and control for important covariates, such as mode of birth and history of mental disorders. However, several important limitations should be addressed when interpreting our results. 

First, our findings lack objective information on previous traumatic experiences and mental health conditions and rely solely on self-reports, which is a common limitation in perinatal studies, particularly in countries without extensive registry-based data. Second, in the question about past traumatic experiences, we did not ask about the timing of the traumatic events; thus, physical and sexual assault might overlap with childhood abuse experiences. Third, 37.5% of women in our sample had clinically significant depressive symptoms according to EPDS, which may bias their recollection of both past traumatic and childbirth experiences. Next, the data collection was completed online, which presents risks of the participants’ mistrust of the researchers and may affect the reliability of their responses. However, the online form of the study contributed to the wide geography of the regions from where the participants came, which is an important factor when collecting data in a country as big as Russia. Nevertheless, another limitation of the online form of the study is its restriction to the participants with access to a device with an Internet connection, which is not available in all regions of Russia. Furthermore, women in our study may also have been more active on social media platforms, which was our primary modality of recruitment. Our sample also consists of self-selected participants, thus presenting the risk of selection bias and lack of women from marginalized and high-risk groups. Finally, relative to the general population, where approximately 40% of adults aged 25–34 have higher education [84], our sample included women who were more educated, with over 90% having a university degree, which limits the generalizability of our findings. Thus, further studies reaching mothers from low-income groups are warranted to obtain a more representative picture of perinatal mental health in Russia.

## 6. Conclusions

A past traumatic experience is a significant risk factor for experiencing childbirth and labor as traumatic, with or without developing PP-PTSD. In particular, child abuse appears to be the strongest predictor of higher distress measured using both the CBiTS scale and subjective rates of traumatic birth experiences. Furthermore, there is a negative cumulative trauma effect, with each additional traumatic experience in the past putting women at increased risk for developing PP-PTSD. While our findings regarding perinatal loss and previous traumatic birth experience are less consistent, taken together, these findings point out the importance of collecting information about women’s past experiences during pregnancy. Therefore, trauma awareness training for midwives, obstetricians, and other caregivers working with women during the perinatal period is essential and trauma-informed practices during childbirth are warranted for the prevention of PP-PTSD. Finally, although we have not seen evidence of the buffering effects of non-medical support during labor for women with past traumatic experiences, the findings indicate its universal protective effect against traumatic birth experiences, particularly with more than one birth support person present at childbirth. Thus, providing continuous support during labor and allowing women to have a birth team of choice may be a simple and cost-effective approach for providing support in all birth settings, minimizing the incidence of PP-PTSD, and, overall, improving the quality of the childbirth experience. 

## Figures and Tables

**Table 1:** Characteristics of the sample (*n* = 2579).

Characteristic		Mean	SD	Range
Age		31.03	4.30	18–46
Time since childbirth		5.49	3.52	0–12
Gestational age at birth		39.57	1.65	25.0–43.0
EPDS		9.09	6.16	0–30
How traumatic did you find your birth?		3.84	2.56	1–10
CBiTS total score (Q3–Q22)		15.79	10.08	0–53
Cumulative traumatic experiences		0.87	1.05	0–7
		**N**	**%**	
Education	Primary	44	1.7%	
	Secondary	185	7.2%	
	Tertiary	2350	91.1%	
Family status	Married	2366	91.7%	
	In relationship	151	5.9%	
	Single	51	2.0%	
SES ^1^	Low-income	263	10.2%	
	Middle-income	1707	66.2%	
	High-income	609	23.6%	
Mode of birth	Vaginal	1882	73%	
	Assisted vaginal	64	2.5%	
	Emergency cesarean	406	15.7%	
	Planned cesarean	227	8.8%	
Region of childbirth facility	Moscow	589	22.8%	
	St. Petersburg	289	11.2%	
	Another region in Russia	1701	66%	
Parity	1	1628	63.1%	
	2	700	27.1%	
	3+	251	9.7%	
Support during labor (yes)		1083	42%	
Mode of support	No support	1496	58%	
	Partner	649	25.2%	
	Doula or Private midwife	227	8.8%	
	Partner + Doula or Private midwife	207	8%	
Previously diagnosed mental disorders	Yes	247	9.6%	
	Unsure	277	10.8%	
	No	2050	79.6%	
Clinically significant symptoms of PPD (EPDS > 10)		967	37.5%	
Clinically significant symptoms of PP-PTSD (according to DSM-5)		528	20.5%	
Traumatic experience of previous childbirth (yes)		515	20%	
Perinatal loss experience (yes)		596	23.1%	
Physical Assault (yes)		205	7.9%	
Sexual Assault (yes)		281	10.9%	
Child abuse (yes)		426	16.5%	
No previous trauma		1191	46.2%	

^1^ Note. SES—Socioeconomic Status; EPDS—The Edinburgh postnatal depression scale; PPD—Postpartum Depression; CBiTS—The City Birth Trauma Scale; PP-PTSD—Postpartum Post-traumatic Stress Disorder; DSM-5—Diagnostic and Statistical Manual of Mental Disorders, 5th edition.

**Table 2:** Associations of the cumulative past traumatic life experiences and support during labor with PP-PTSD measured as continuous CBiTS scores and binary outcome compatible with DSM-5 criteria and the subjective rates of traumatic birth experiences.

Predictor	Cumulative Traumatic Experiences (0–7)				Support during Labor (Yes/No)			
Outcome	B/OR *	SE	95% CI	*p*-Value	B/OR	SE	95% CI	*p*-Value
PTSD symptoms according to CBiTS continuous scale (0–53)	1.88	0.18	1.52–2.24	<0.001	−0.89	0.40	−1.67; −0.10	0.027
Clinically significant PTSD according to DSM-5 (yes/no)	1.26	0.05	1.15–1.38	<0.001	0.73	0.11	0.58; 0.90	0.004
Subjective rates of traumatic birth experience (0–10)	0.18	0.05	0.09–0.27	<0.001	−0.074	0.10	−0.93; −0.54	<0.001

* Note: B refers to unstandardized regression coefficient from multiple regression model; OR refers to odds ratio from bivariate logistic regression; SE refers to standard error; 95% CI refers to 95% confidence interval. All models are adjusted for maternal age at the time of childbirth, level of education, family status, SES, history of mental disorders, gestational age at birth, time since childbirth, and mode of birth.

## Data Availability

We are ready to provide an anonymized dataset, syntaxes, and the survey form (in Russian) by request.

## Supplementary Materials

The following supporting information can be downloaded at: https://www.mdpi.com/article/10.3390/ijerph20116048/s1, Table S1: Associations between PP-PTSD measured continuously and categorically and the subjective rates of traumatic birth experience and support during labor in the subsamples stratified by the absence/presence of past traumatic experiences.
