# Supplementary material for: Past Traumatic Life Events, Postpartum PTSD, and the Role of Labor Support
Source: Int J Environ Res Public Health. 2023 Jun 4;20(11):6048. doi: 10.3390/ijerph20116048 (PMC10252538; doi:10.3390/ijerph20116048)
Supplement: Supplementary file 1 [file ijerph-20-06048-s001.zip › ijerph-2331911-supplementary.pdf]

Table S1. Associations between PP-PTSD measured continuously and categorically and the subjective rates of traumatic birth experience and support during labour in the subsamples stratified by the absence/presence of past traumatic experiences.

Outcome:	PTSD symptoms according to CBiTS continuous scale (0-53)				Clinically significant PTSD according to DSM-5 (yes/no)				Subjective rates of traumatic birth experience (0-10)			
Predictor: Support during labour in the presence of	B/OR	SE	95% CI	p-value	B/OR	SE	95% CI	p-value	B/OR	SE	95% CI	p-value
No past trauma	-0.82	0.57	-1.95; 0.30	0.15	0.76	0.19	0.53; 1.09	0.13	-0.75	0.15	-1.04; -0.38	<0.001
At least one traumatic event in the past	-1.08	0.54	-2.15; -0.01	0.047	0.70	0.14	0.53; 0.92	0.010	-0.71	0.14	-1.01; -0.47	<0.001
No physical assault in the past	-0.84	0.41	-1.65; -0.03	0.042	0.72	0.12	0.57; 0.90	0.004	-0.74	0.10	-0.94; -0.54	<0.001
Physical assault in the past	-2.04	1.54	-5.07; 0.99	0.19	0.85	0.38	0.40; 1.80	0.67	-0.80	0.38	-1.55; -0.05	0.038
No sexual assault in the past	-0.93	0.42	-1.76; -0.10	0.027	0.70	0.12	0.55; 0.88	0.002	-0.73	0.11	-0.93; -0.52	<0.001
Sexual assault in the past	-0.32	1.25	-2.78; 2.15	0.80	1.15	0.33	0.61; 2.18	0.67	-0.81	0.32	-1.44; -0.18	0.011
No child abuse in the past	-0.92	0.43	-1.77; -0.078	0.032	0.76	0.13	0.59; 0.97	0.027	-0.74	0.11	-0.95; -0.53	<0.001
Child abuse in the past	-1.21	1.0	-3.17; 0.75	0.23	0.58	0.24	0.36; 0.93	0.023	-0.79	0.26	-1.30; -0.29	0.002
No perinatal loss in the past	-0.69	0.46	-1.59; 0.21	0.13	0.75	0.13	0.58; 0.97	0.027	-0.70	0.12	-0.93; -0.48	<0.001

Perinatal loss in the past	-1.61	0.82	-3.22; 0.002	0.050	0.65	0.22	0.42; 1.002	0.051	-0.85	0.20	-1.24; -0.45	<0.001
No previous childbirth	-0.30	0.52	-1.31; 0.72	0.57	0.87	0.13	0.67; 1.13	0.31	-0.73	0.13	-0.98; -0.48	<0.001
Previous childbirth not traumatic	-1.48	0.91	-3.27; 0.31	0.10	0.56	0.38	0.26; 1.18	0.13	-0.77	0.25	-1.25; 0.29	0.002
Previous traumatic birth experience	-1.87	0.88	-3.61; -0.13	0.035	0.53	0.27	0.31; 0.90	0.019	-0.53	0.22	-0.97; -0.10	0.015

Note: B refers to unstandardized regression coefficient from multiple regression model; OR refers to odds ratio from bivariate logistic regression; SE refers to standard error; 95% CI refers to 95% confidence interval.
All models are adjusted for maternal age at the time of childbirth, level of education, family status, socioeconomic status, history of mental disorders, gestational age at birth, time since the childbirth, and mode of birth.
